# Albert Fry

**Published:** 1903-09

**Authors:** 


### ALBERT FRY.

We regret that the occasion has arisen for comment upon the death of Mr. Albert Fry, who was for nearly twenty years Chairman of the Council of Bristol University College. He died at his residence in Clifton on April 22nd, 1903, in his 72nd year. Although Mr. Fry had been in failing health for several months, he continued to preside over the meetings of the Council and the local Executive Committee until a short time before his death. Mr. Fry was by profession an engineer, and for many years Managing Director of the Bristol Wagon and Carriage Company. He possessed a large and varied experience of men and things, great business experience and capacity, and a sound and singularly impartial judgment. He was in many ways an important citizen; but we confine our remarks to his connection with University College, and it would be difficult to over-estimate the value of the services he rendered to this Institution. Mr. Fry was an ideal Chairman of the Council. His good influence made itself felt in every department of the College, and was a great factor in producing that harmonious working of the complex organization which is so essential to the progress and prosperity of such an Institution.

During Mr. Fry's tenure of office the College had considerably increased in size and importance; he had the satisfaction of seeing the Engineering wing, the new Medical buildings and the large Hall and Library begun and completed, and but for lack of the necessary funds the whole building as originally contemplated would have been finished—no mean feat when the pecuniary difficulties under which the College has always laboured are considered, and which were overcome so far as they have been overcome by, in large part, the energy and influence of Mr. Fry.

Mr. Fry always took a deep interest in the Medical Department of the College, was one of the governing body of the Medical School from the year 1882, when he succeeded Mr. Nonus Budd, down to the incorporation of the Medical School with the College in 1892. When the new Medical Wing was in course of construction, he gave in many ways much valuable assistance with the plans, besides being a generous contributor to the Building Fund.

